# Segmental Distribution of Hepatocellular Carcinoma in Cirrhotic Livers

**DOI:** 10.3390/diagnostics12040834

**Published:** 2022-03-29

**Authors:** Matteo Renzulli, Nicolò Brandi, Anna Pecorelli, Luigi Vincenzo Pastore, Alessandro Granito, Giuseppe Martinese, Francesco Tovoli, Mario Simonetti, Elton Dajti, Antonio Colecchia, Rita Golfieri

**Affiliations:** 1 Department of Radiology, IRCCS Azienda Ospedaliero-Universitaria di Bologna, Via Albertoni 15, 40138 Bologna, Italy; pecorelli.anna@gmail.com (A.P.); luigi.pastore3@studio.unibo.it (L.V.P.); giuseppe.martinese@studio.unibo.it (G.M.); mario.simonetti2@studio.unibo.it (M.S.); rita.golfieri@unibo.it (R.G.); 2 Division of Internal Medicine, IRCCS Azienda Ospedaliero-Universitaria di Bologna, 40138 Bologna, Italy; alessandro.granito@unibo.it (A.G.); francesco.tovoli2@unibo.it (F.T.); 3 Department of Medical and Surgical Sciences (DIMEC), IRCCS, University of Bologna, Via Massarenti 9, 40138 Bologna, Italy; e_dajti17@hotmail.com; 4 Unit of Gastroenterology, Borgo Trento University Hospital of Verona, 25122 Verona, Italy; antonio.colecchia@aovr.veneto.it

**Keywords:** hepatocellular carcinoma, liver, cirrhosis, neoplasms, liver imaging

## Abstract

Background: To evaluate the segmental distribution of hepatocellular carcinoma (HCC) according to Couinaud’s anatomical division in cirrhotic patients. Methods: Between 2020 and 2021, a total of 322 HCC nodules were diagnosed in 217 cirrhotic patients who underwent computed tomography (CT) or magnetic resonance imaging (MRI) for the evaluation of suspicious nodules (>1 cm) detected during ultrasound surveillance. For each patient, the segmental position of the HCC nodule was recorded according to Couinaud’s description. The clinical data and nodule characteristics were collected. Results: A total of 234 (72.7%) HCC nodules were situated in the right lobe whereas 79 (24.5%) were detected in the left lobe (*p* < 0.0001) and only 9 nodules were in the caudate lobe (2.8%). HCC was most common in segment 8 (*n* = 88, 27.4%) and least common in segment 1 (*n* = 9, 2.8%). No significant differences were found in the frequencies of segmental or lobar involvement considering patient demographic and clinical characteristics, nodule dimension, or disease appearance. Conclusions: The intrahepatic distribution of HCC differs among Couinaud’s segments, with segment 8 being the most common location and segment 1 being the least common. The segmental distribution of tumour location was similar to the normal liver volume distribution, supporting a possible correlation between HCC location and the volume of hepatic segments and/or the volumetric distribution of the portal blood flow.

## 1. Introduction

Hepatocellular carcinoma (HCC) is currently the fifth most common malignancy worldwide, representing the second leading cause of cancer-related deaths and the first for cirrhotic patients [1,2]. Furthermore, its incidence has constantly increased in recent years and HCC is now considered to be the fastest growing cause of cancer-related death in the United States [3].

Only approximately 20% of HCCs are diagnosed in the very early and early stages when treatments, such as liver transplantation, ablation and surgical resection, can guarantee a high 5-year survival rate. On the contrary, the majority of HCC patients are in the intermediate and/or advanced tumoural stages at presentation and are therefore unsuitable for these treatments. They require instead transarterial chemoembolisation (TACE), radioembolisation, or systemic therapies, which are considered effective treatments, but they yield a lower overall survival rate than the treatments mentioned above [4,5,6,7]. However, progressive improvements in imaging techniques have appeared in recent years, and together with new schemes for surveillance programs, are overcoming the limitations of ultrasound (US) to detect an ever-growing number of lesions, the majority of which are now at the very early and early stages [8,9,10,11]. These new strategies will improve patient prognoses, thanks to the possibility of referring patients to the best treatment.

The anatomical division described by Couinaud [12] is currently the most widely utilized system for describing functional liver anatomy since it is best adapted for liver surgery. In fact, the precise delineation of each segment based on its own dual vascular inflow, biliary drainage and lymphatic drainage allows a safe and independent surgical resection, minimising blood loss and preserving the remnants of functional parenchyma [13]. According to this classification, which is under-recognised in the scientific literature, tumour location is a potential factor that may influence patient prognoses. The HCC location may have an impact on both the diagnostic strategy (e.g., whether or not to perform a biopsy) and the choice of the most effective therapy (e.g., identifying those locations that are unfavourable for percutaneous treatments) [14]. Furthermore, tumours are considered to have a favourable location when they occur in the anterolateral segments (i.e., segments 2, 3, 4b, 5 and 6) and an unfavourable location when they occur in the posterosuperior segments (i.e., segments 1, 4a, 7 and 8) [15]. For example, HCCs located in segment 4a (i.e., in a subcardiac location) are associated with a higher risk of ablation technical failure, essentially due to the continuous movement caused by cardiac motion during treatment [16]. Tumour locations in liver segments 4a, 8 and, particularly, 7 represent independent predictors of local recurrence after ablation due to a greater difficulty of probe positioning [17]. Moreover, the risk of microvascular invasion is 3.5 times higher when the HCC is located in segment 8 as compared to the other segments, using imaging for its preoperative identification as a prognostic factor [3]. A left-sided location of HCC is a significant preoperative predictor of a poor prognosis in patients who undergo liver resection due to the higher frequency of hematogenous metastasis [18]. In addition, the overall survival and local tumour progression rates were poorer for tumours located in segment 2, probably due to being adjacent to the diaphragm and heart and the consequent difficulty in detecting the lesion under US guidance [19]. Finally, other locations are associated with positive outcomes: for example, segments 6 and 7 were strongly associated with a complete response to TACE in patients with unresectable HCC [20]. 

When analysing different tumours other than HCC, such as colorectal cancer, the most frequent cancer of the gastrointestinal system, it is well known that there are differences in the histological characteristics, and therefore, the prognosis according to the location of the tumour. For example, right-sided colon cancers (proximal to the splenic flexure) and left- sided colon cancers (distal to the splenic flexure) are two distinct diseases with different clinical presentations, treatment responses and survival outcomes [14,21]. Moreover, growing evidence suggests that these two different colon cancers may even be considered distinct entities due to their varying carcinogenic pathways and DNA mutations. In fact, right-sided colon cancers have been more commonly associated with microsatellite instability, BRAF mutations and DNA hypermethylation in CpG islands, whereas left-sided cancers frequently demonstrate chromosomal instability, loss of heterozygosity and KRAS mutations [22].

Consequently, it would be interesting to investigate whether HCC shows a preferential location of development in the liver, which could allow radiologists to carry out a tailored evaluation of radiological imaging based on these data. In addition, it would be equally interesting to assess whether there are some factors that may influence the HCC site of origin.

Unfortunately, there are currently only a few studies in the literature regarding the anatomical preference for the appearance of HCC, all with small study populations [16,23,24]. Moreover, the clinical and radiological features that may correlate with a specific laterality or location have not yet been investigated.

The aim of the present study was to evaluate the segmental distribution of HCC according to Couinaud’s anatomical division in a large series of cirrhotic patients, analysing any possible clinical and radiological features that may correlate with a specific anatomical localisation. 

## 2. Materials and Methods

This was a prospective study approved by the local institutional review board (protocol number: 216/2018/AOUBo). Written informed consent was obtained from all patients, and the study was conducted in compliance with the Declaration of Helsinki for clinical studies. The current study was not supported by any industry or pharmaceutical company.

### 2.1. Patient Characteristics and Imaging Technique

Between 2020 and 2021, a total of 1067 patients with cirrhosis underwent computed tomography (CT) or magnetic resonance imaging (MRI) at the authors’ tertiary centre for the evaluation of suspicious nodules (>1 cm) detected during ultrasound surveillance. 

Contrast-enhanced CT and MRI were performed according to the standards of reference recommended by international guidelines [5,25].

Three hundred and sixty-three patients had a diagnosis of HCC, based on the European Association for the Study of the Liver (EASL) criteria for nodules >1 cm in patients with a high risk of developing HCC [26], and were prospectively enrolled.

Patients with a previous history of HCC and/or incomplete records (*n* = 146) were excluded. 

Clinical data, such as age, gender, Child–Pugh score and hepatic venous pressure gradient (HVPG), were collected.

### 2.2. Image Analysis

Locating the HCCs and radiological diagnoses were carried out independently using CT or MRI findings by radiologists with more than fifteen years of experience in liver imaging. For each patient, the segmental position of the HCC nodules was recorded according to Couinaud’s description [27]. According to a previous study [28], when a tumour was located across two or more Couinaud’s segments, the location of the tumour centre was defined as the representative location. The right and left lobes were divided by Cantlie’s line, a vertical plane that extends from the inferior vena cava posteriorly to the middle of the gallbladder fossa anteriorly and contains the middle hepatic vein; the left hemiliver was therefore considered to include segments 2, 3 and 4 while the right hemiliver included segments 5, 6, 7 and 8. The caudate lobe receives blood vessels and biliary tributaries from both the right and left hemilivers; it is divided from the right lobe by an imaginary line perpendicular to the right portal vein bifurcation, and from the left lobe by an imaginary line between the fissure for the ligamentum venosum and the right portal vein.

Patients were classified as having uninodular (1 nodule), oligonodular (2–3 nodules) or multinodular (>3 nodules) disease based on the number of nodules identified by imaging according to Vitale et al. [29]. In addition, the patients were divided on the basis of whether they were within or beyond the Milan criteria.

Data relating to liver stiffness, spleen stiffness and spleen diameter were collected with the intent of investigating a possible role for the portal blood flow and portal hypertension in the distribution of HCC nodules. In particular, the splenic diameter was measured in terms of its width in axial images (normal threshold of 10.5 cm), which represents the most sensitive and specific single measurement for the detection of splenomegaly in cirrhotic patients [30].

### 2.3. Statistical Analysis

The continuous variables are expressed as the mean ± standard deviation (SD), and the categorical variables as the number of cases and proportions. Variable distribution was assessed using the Kolmogorov–Smirnov test, and the continuous variables were compared using an analysis of variance (ANOVA). The categorical variables were compared using the chi-square test with Yates’ correction. A two-tailed *p* < 0.05 was considered statistically significant. All statistical analyses were carried out using the SPSS 21.0 statistical package (SPSS Incorporated, Chicago, IL, USA).

## 3. Results

**Figure 1:**
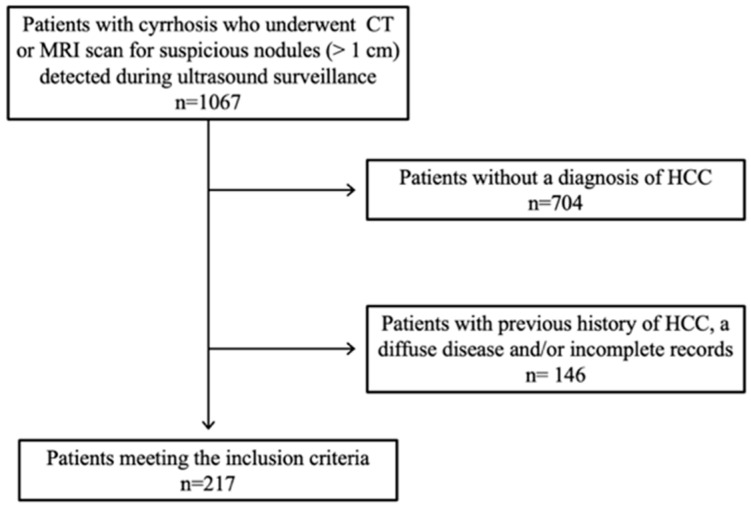
Flow diagram of patient selection for the study.

During the study period, 217 patients were diagnosed as having a total of 322 HCC nodules (Figure 1). The mean age of the patients was 65 years (±11 years) and there were 41 women (18.9%) in this cohort. 

The majority of the nodules (169 nodules/52.5%) were detected in patients with cirrhosis related to chronic viral hepatitis, followed by alcohol abuse and nonalcoholic steatohepatitis (NASH) (61 nodules/18.9% and 45 nodules/14%, respectively). 

At the time of diagnosis, the majority of the nodules (255/82%) had developed in patients with well-compensated liver function (defined as Child–Pugh class A), while the minority arose within the setting of moderate compensated liver function (50 nodules/16.1% Child–Pugh class B) or decompensated cirrhotic patients (6 nodules/1.9% Child–Pugh class C patients). 

**Figure 2:**
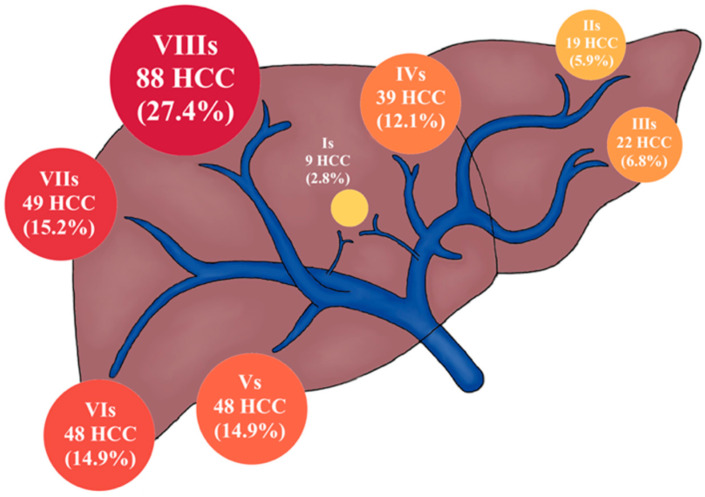
The distribution of HCC locations by liver segment; the size of the circles in the image represents the percentage of total HCC nodules.

**Figure 3:**
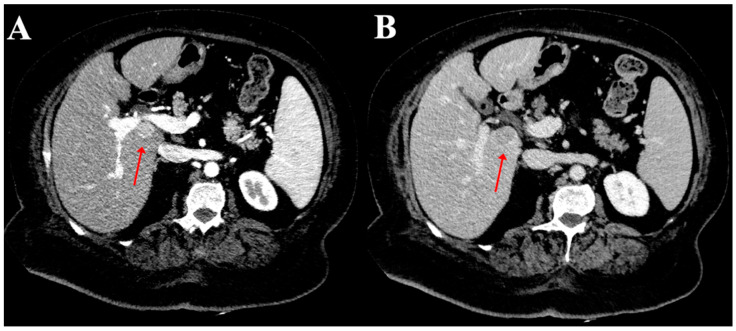
Computed tomography of the axial planes of an HCC nodule in the segment 1 (red arrows) showing hyperenhancement in the arterial phase (**A**) and wash-out in the portal phase (**B**).

**Figure 4:**
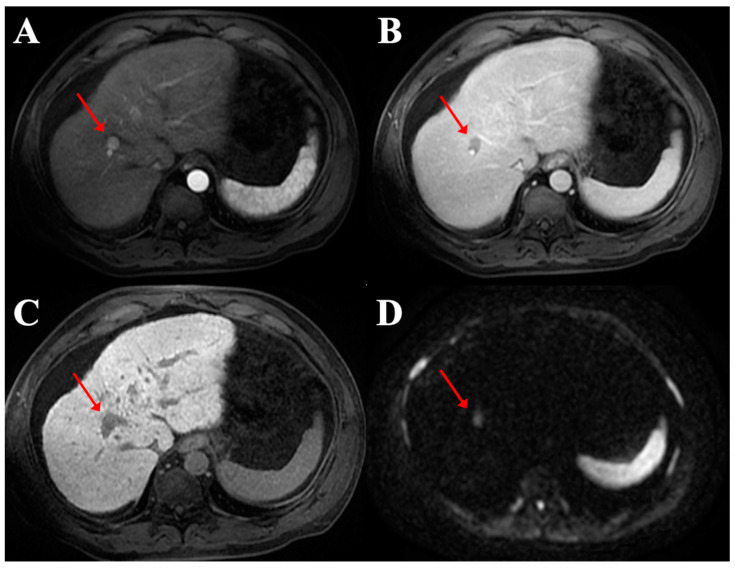
Magnetic resonance imaging of the axial planes of a HCC nodule (red arrows) in segment 8 showing hyperenhancement in the arterial phase (**A**), wash-out in the portal phase (**B**), hypointensity in the hepato-biliary phase (**C**) and diffusion restriction on the diffusion weighted imaging (DWI) using b-values of 800 s/mm^2^ (**D**).

A total of 234 HCC nodules were situated in the right lobe (72.7%) whereas 79 were detected in the left lobe (24.5%). This difference was statistically significant (*p* < 0.0001) and produced a ratio of almost 3:1. Only nine nodules were located in the caudate lobe (2.8%). The distribution of HCC in each liver segment is presented in Figure 2, where it was most common in segment 8 (88/27.4%) (Figure 3) and least common in segment 1 (9/2.8%) (Figure 4).

No significant differences were found in the frequencies of segmental or lobar involvement when considering demographic data, Child–Pugh score and cirrhotic etiology (Table 1 and Table 2).

Considering the number of nodules, the uninodular appearance of HCC was slightly less common in the left lobe as compared to the right and caudate lobes, however this was not statistically significant (41.8% vs. 50% and 55.6%, respectively, *p* = 0.755). Oligonodular and multinodular disease was roughly comparable for all subgroups. Moreover, no significant differences in anatomical distribution were found between patients within and those outside the Milan Criteria (Table 1 and Table 2).

The mean size of the HCC nodules was 3.1 cm (±2.8 cm). Approximately half of the tumours (*n* = 158/49.1%) were ≤2 cm, 62 (19.2%) were >2 cm but ≤3 cm, 52 were >3 cm but ≤5 cm (16.2%) and 50 (15.5%) were >5 cm. When considering nodule size, no significant differences were evident in the frequencies of segmental or lobar involvement.

No significant correlation was found between HCC location, hepatic and spleen stiffness, and spleen diameter.

## 4. Discussion

The location of HCC is currently considered to be an important factor influencing both diagnostic strategy and therapeutic options. In fact, when a lesion is situated in the deeper segments, such as 4b, 7 and 8, the rate of successful bioptic sampling can be diminished due to the technical difficulties in reaching these less accessible locations [31,32]. Similarly, the precise definition of a tumour site allows its adjacency to large vessels, the biliary tree, the liver capsule or other important tissues, such as the diaphragm or gastrointestinal tracts, to be evaluated for the most appropriate and effective therapeutic choice, whether it is percutaneous or surgical [3,15,16,17,18,19,20]. However, in addition to these technical and prognostic implications, only a few studies have analysed the spatial distribution of HCC with reference to Couinaud’s segments [28]. 

The present study showed that the majority of HCC nodules arose in the right lobe (72.7%%) with a ratio of predisposition for right-left lobe involvement of almost 3:1, a result that is consistent with that found in the literature (57–68%) [18,33,34]. Moreover, a particularly interesting result was that only 2.8% of the nodules were in the caudate lobe.

The segmental distribution of HCC according to Couinaud’s division showed that segment 8 represented the most common location (27.4%), whereas segment 1 was the least common (2.8%). Although liver volumes were not evaluated in this study, the segmental distribution of HCC was proportional to the normal segmental (and lobar) volumes previously reported in the literature [19], which was more evident compared to a previous study [28] in which, on the contrary, segment 6 was documented as the most common location. In the present study, segment 8 represented the most common site of HCC location, which is in line with the fact [19,28] that the largest volume is found in segment 8 as compared to the other segments. Consequently, segment 1, which is the smallest liver segment, represents the least common site of HCC.

Interestingly, the results were not statistically significant when location was analysed in terms of uninodular, oligonodular and multinodular disease, or regarding patients inside versus outside the Milan criteria, probably because non-uninodular disease arises with more frequency and perhaps even earlier in the segments with a larger volume.

The evidence that the frequency of segmental localisation is correlated with the volume of the segments is also supported by the previous results of Sakuraoka et al. [18]. In fact, HCC recurrence was higher in left-sided resections as compared to right-sided resections, possibly due to the larger size of the liver remnant and the smaller resection margin.

Assuming that there is a correlation between HCC localisation and the volume of hepatic segments, it is important to remember that cirrhosis can lead to segmental anatomic changes, including atrophy of the left medial segment and right lobe, and compensatory hypertrophy of the left lateral segment and caudate lobe [35,36]. Moreover, these changes may slightly differ among the various aetiologies of liver cirrhosis [37]. For example, hypertrophy of the left lateral segment has, for the most part, been found in hepatitis B cirrhosis while hypertrophy of the caudate lobe has been associated with alcoholic cirrhosis [28,35]. Moreover, as reported by Zhou et al. [38], even the severity of liver cirrhosis may influence hepatic segmental changes, with absolute hypertrophy of the left lateral segment in Child–Pugh A and B as compared to Child–Pugh C subjects, and absolute enlargement of the caudate lobe in Child–Pugh A as compared to Child–Pugh B and C subjects. For all of these reasons, a possible correlation between HCC segmental distribution and the aetiology of cirrhosis, as well as with the Child–Pugh score and cirrhosis decompensation, was investigated but no significant differences emerged in the present study. Therefore, it is possible to assume that the small variations occurring regarding cirrhotic changes are probably irrelevant to the development of the tumour in one segment/lobe rather than another, probably because these volumetric differences are not significant enough.

Although studies regarding HCC localisation are scarce, those that have analysed the distribution of colorectal metastases are numerous and unanimously affirm that metastatic disease affects the right lobe more than the left lobe (63–70% vs. 27–30%) [39]. Therefore, it can be hypothesised that there is a parallel between colorectal metastases and HCC distribution, so much so that a correlation with the hepatic lobe volume has also been assumed in studies regarding metastasis location [40]. Moreover, several authors have claimed that the portal vein and blood flow play a key role in the segmental distribution of liver metastasis [40]. The concept of streamlining suggests that venous blood from the right and transverse colon is drained by the superior mesenteric vein, which predominantly travels along the right side of the portal vein and into the right hepatic lobe; on the contrary, the venous drainage of the left colon and the rectum is sustained by the inferior mesenteric vein, which usually drains into the splenic vein and travels predominantly into the left lobe [41]. Therefore, it has been hypothesised that metastases arising from the right-sided colon would more frequently invade the right lobe while metastases of the left-sided colon and rectal carcinomas could be more frequent in the left hemiliver [41]. However, several studies [42,43] have demonstrated that the greater metastatic involvement of the right hemiliver did not change when patients were categorised according to whether they had right colonic or left colonic rectal primary tumours. The evidence for the greater prevalence of metastases in the right hepatic lobe, and also in left-sided colon carcinomas, could be explained by the fact that the inferior mesenteric vein terminates variably in either the splenic or the superior mesenteric vein, thus contributing to the variation in distribution. More importantly, it has been demonstrated that the liver is heterogeneously perfused, and the right lobe receives a higher portal flow as compared to the left lobe, essentially due to the anatomical appearance of the main portal vein [44]. In fact, in the majority of cases (70.9–86.2%, according to Cheng et al. [45]), the left portal vein is separated from the main portal vein at an acute angle while the right portal vein is the direct continuation of it, thus guaranteeing a greater blood flow.

Similar to colorectal metastases, the predisposition of HCC to develop in the right lobe could also be linked to its greater portal blood flow. In the same way that the portal vein can deliver cancer cells to the liver from colon-rectal sites, it may also deliver intestinal microbial metabolites (such as lipopolysaccharide, bacterial DNA and peptidoglycan) that can lead to the production of inflammatory cytokines and chemokines, causing hepatocyte damage, promoting fibrosis and, ultimately, HCC development [46,47].

Regarding patients with portal hypertension, portal venous inflow is decreased, and the consequent hepatic arterial/portal venous inflow ratio is considerably affected, thus leading to an elevated hepatic perfusion index. With the aim of investigating a possible role for portal blood flow in the distribution of HCC nodules, data relating to the HVPG, liver stiffness and spleen stiffness were collected in the present study in order to evaluate any flow variations [48,49,50,51]. The latest literature [52,53] has suggested that both liver and spleen stiffness correlate with the HPVG and are impacted by portal hypertension. However, no significant correlation was found between HCC location and hepatic and spleen stiffness, or with spleen diameter. Even if portal hypertension is correlated with HCC onset due to an increased arterial perfusion index, these vascular changes involve the entire liver parenchyma homogenously, and thus, will not influence HCC location.

The present results, if confirmed by further studies, could improve the daily clinical practice for managing HCC patients. In fact, in the era of tailored medicine, HCC location could help both hepatologists and interventional radiologists in their decision-making strategy for the most effective treatment for each patient according to tumour location. In the field of hepatology, the choice of chemoembolization repetition and the optimum number of chemoembolization sessions before switching to another treatment or best supportive care still represent major issues [7]. The effectiveness of this treatment in some cases may be low due to the tumour’s location in a segment with small arteries that are difficult to catheterize selectively, such as segment 1. This may explain why many proposed scores, such as the ART score, are not objective tools for guiding chemoembolization re-treatment in all instances and populations [7]. In the era of combined therapies, an understanding of the disadvantaged locations of HCC could help guide decisions on the association of a locoregional treatment, such as chemoembolization, with systemic therapy. Furthermore, many other studies could be planned to understand the best treatment strategies for HCC based on its location. 

The present study had several limitations. First, it included patients from a single medical centre. Second, the hepatic segmental volume distributions for the patients included in the present study were not recorded, and our considerations were made in accordance with the literature regarding normal livers and no history of liver disease. Finally, the present cohort was evaluated with MRI and CT scans, with the latter being less sensitive; however, the current guidelines for the management of HCC [25] states that a single modality CT or MRI is sufficient when radiological hallmark features are present without the need for another imaging technique for confirmation. 

Considering the large population included in the present study, as compared to previous studies, the present results will contribute significantly to the literature regarding intrahepatic HCC distribution. Moreover, one of the strengths of the present study is the heterogeneity of our cohort since, contrary to previous studies that analysed only specific subgroups of patients (those who underwent transplant, surgical resection and/or percutaneous treatments), it represents a faithful reproduction of the cirrhotic population under surveillance. Finally, several previous studies [18,19] excluded segment 1 (the caudate lobe) from all calculations due to its dual blood supply, which provides a less realistic and comprehensive view of this topic.

## 5. Conclusions

The present study showed that the intrahepatic distribution of HCC differed among Couinaud’s segments, with segment 8 as the most common location and segment 1 as the least common. Moreover, Couinaud’s segmental distribution of tumour location was very similar to the normal volume distribution of the liver, thus supporting a possible correlation between HCC location and the volume of the liver segments and/or the volumetric distribution of the portal blood flow.

## Figures and Tables

**Table 1:** Patient characteristics according to the lobar location of HCC.

	Liver Lobe			
	Left	Right	Caudate	*p*
Nodule Total (*n* = 322)	79 (24.5)	234 (72.7)	9 (2.8)	
Gender *				
Male	62 (78.5)	198 (84.6)	7 (77.8)	0.419
Female	17 (21.5)	36 (15.4)	2 (22.2)	
Age ^§^	64 (±11.9)	65 (±10.7)	62 (±8.8)	0.570
Etiology of cirrhosis				
Viral	36 (45.6)	128 (54.7)	5 (55.6)	0.412
Alcohol	21 (26.6)	38 (16.2)	2 (22.2)	
NASH-NAFLD	8 (10.1)	36 (15.4)	1 (11.1)	
Others	14 (17.7)	33 (14.1)	1 (11.1)	
Type of HCC				
Monofocal	33 (41.8)	117 (50)	5 (55.6)	0.755
Oligonodular	15 (19)	37 (15.8)	1 (11.1)	
Multinodular	31 (39.2)	80 (34.2)	3 (33.3)	
Milan criteria				
Milan in	64 (81)	197 (84.2)	8 (88.9)	0.731
Milan out	15 (19)	37 (15.8)	1 (11.1)	
Nodule size				
≤2 cm	33 (41.8)	119 (50.9)	6 (66.7)	0.213
>2 cm but ≤3 cm	20 (25.3)	42 (17.9)	0	
>3 but ≤5 cm	14 (17.7)	37 (15.8)	1 (11.1)	
>5 cm	12 (15.2)	36 (15.4)	2 (22.2)	
Spleen diameter				
≤12 cm	44 (55.7)	118 (50.4)	4 (44.4)	0.657
>12 cm	35 (44.3)	116 (49.6)	5 (55.6)	
Spleen stiffness				
≤61 kPa	37 (46.8)	100 (43.5)	3 (33.3)	0.673
>61 kPa	42 (53.2)	134 (56.5)	6 (66.7)	
Liver stiffness				
≤29 kPa	37 (46.8)	85 (36.3)	5 (55.6)	0.154
>29 kPa	42 (53.2)	149 (63.7)	4 (44.4)	
Nodules in Child–Pugh score				
Class A (5–6)	63 (79.7)	192 (82.1)	8 (88.9)	0.559
Class B (7–9)	14 (17.7)	38 (16.2)	1 (11.1)	
Class C (10–15)	2 (2.5)	4 (1.7)	0	
HPVG				
<12 mmHg	38 (48.1)	93 (39.7)	6 (66.7)	0.143
≥12 mmHg	41 (51.9)	141 (61.3)	3 (33.3)	

Unless indicated otherwise, data are the number of nodules, with percentages in parentheses. * Data are the number of patients, with percentages in parentheses. ^§^ Data are continuous variables, reported as the mean with the standard deviation in parentheses.

**Table 2:** Patient characteristics according to the segmental location of HCC.

	Liver Segment								
	S1	S2	S3	S4	S5	S6	S7	S8	*p*
Nodule Total (*n* = 322)	9 (2.8)	19 (5.9)	22 (6.8)	39 (12.1)	48 (14.9)	48 (14.9)	49 (15.2)	88 (27.4)	
Gender *									
Male	7 (77.8)	16 (84.2)	16 (72.7)	31 (79.5)	43 (89.6)	41 (85.4)	39 (79.6)	74 (84.1)	0.743
Female	2 (22.2)	3 (15.8)	6 (27.3)	8 (20.5)	5 (10.4)	7 (14.6)	10 (20.4)	14 (15.9)	
Age ^§^	62 (±8.8)	66 (±9.5)	66 (10)	62 (±13.6)	66 (±10.4)	65 (±12)	65 (±10)	66 (±10.6)	0.809
Etiology of cirrhosis									
Viral	5 (55.6)	11 (57.9)	10 (45.4)	16 (41.1)	32 (66.7)	26 (54.2)	24 (49)	45 (51.1)	0.771
Alcohol	2 (22.2)	4 (21)	6 (27.3)	11 (28.2)	9 (18.7)	5 (10.4)	10 (20.4)	14 (15.9)	
NASH-NAFLD	1 (11.1)	1 (5.3)	2 (9.1)	5 (12.8)	4 (8.3)	9 (18.7)	7 (14.3)	16 (18.2)	
Others	1 (11.1)	3 (15.8)	4 (18.2)	7 (17.9)	3 (6.3)	8 (16.7)	8 (16.3)	13 (14.8)	
Type of HCC									
Monofocal	5 (55.6)	10 (52.6)	9 (40.9)	14 (35.9)	20 (41.7)	25 (52.1)	26 (53.1)	46 (52.3)	0.860
Oligonodular	1 (11.1)	2 (10.5)	6 (27.3)	7 (17.9)	11 (22.9)	7 (14.6)	6 (12.2)	13 (14.8)	
Multinodular	3 (33.3)	7 (36.8)	7 (31.8)	18 (46.2)	17 (35.4)	16 (33.3)	17 (34.7)	29 (33)	
Milan criteria									
Milan in	8 (88.9)	17 (89.5)	16 (72.7)	32 (82.1)	37 (77.1)	41 (85.4)	43 (87.8)	75 (85.2)	0.660
Milan out	1 (11.1)	2 (10.5)	6 (27.3)	7 (17.9)	11 (22.9)	7 (14.6)	6 (12.2)	13 (14.8)	
Nodule size									
≤2 cm	6 (66.7)	6 (31.6)	12 (54.5)	15 (38.5)	23 (47.9)	24 (50)	30 (61.2)	42 (47.7)	0.294
>2 but ≤3 cm	0	7 (36.9)	5 (22.8)	8 (20.5)	7 (14.6)	9 (18.7)	6 (12.3)	20 (22.8)	
>3 but ≤5 cm	1 (11.1)	4 (21)	2 (9.1)	9 (23.1)	13 (27.1)	7 (14.6)	5 (10.2)	11 (12.5)	
>5 cm	2 (22.2)	2 (10.5)	3 (13.6)	7 (17.9)	5 (10.4)	8 (16.7)	8 (16.3)	15 (17)	
Spleen diameter									
≤12 cm	4 (44.4)	12 (63.2)	13 (59.1)	20 (51.3)	23 (47.9)	26 (54.2)	21 (42.6)	47 (53.4)	0.828
>12 cm	5 (55.6)	7 (36.8)	9 (40.9)	19 (48.7)	25 (52.1)	22 (45.8)	28 (57.1)	41 (46.6)	
Spleen stiffness									
≤61 kPa	3 (33.3)	10 (52.6)	11 (50)	17 (43.6)	16 (33.3)	21 (43.7)	19 (38.8)	43 (48.9)	0.673
>61 kPa	6 (66.7)	9 (47.4)	11 (50)	22 (56.4)	32 (66.7)	27 (56.2)	30 (61.2)	45 (51.1)	
Liver stiffness									
≤29 kPa	5 (55.6)	9 (47.4)	11 (50)	18 (46.2)	15 (31.2)	20 (83.3)	15 (30.6)	34 (38.6)	0.504
>29 kPa	4 (44.4)	10 (52.6)	11 (50)	21 (53.8)	33 (68.8)	28 (58.3)	34 (69.4)	54 (61.4)	
Nodules in Child–Pugh score									
Class A (5–6)	8 (88.9)	13 (68.5)	16 (72.7)	35 (89.7)	42 (87.5)	39 (81.3)	37 (75.5)	73 (83)	0.577
Class B (7–9)	1 (11.1)	5 (26.2)	6 (27.3)	3 (7.7)	6 (12.5)	7 (14.6)	10 (20.4)	15 (17.0)	
Class C (10–15)	0	1 (5.3)	0	1 (2.5)	0	2 (4.2)	2 (4.1)	0	
HPVG									
<12 mmHg	6 (66.7)	10 (52.6)	14 (63.6)	15 (38.5)	21 (43.7)	23 (47.9)	14 (28.6)	34 (38.6)	0.092
≥12 mmHg	3 (33.3)	9 (47.4)	8 (36.4)	24 (61.5)	27 (56.2)	25 (52.1)	35 (71.4)	54 (61.4)	

Unless indicated otherwise, data are the number of nodules, with percentages in parentheses. * Data are the number of patients, with percentages in parentheses. ^§^ Data are continuous variables, reported as the mean with the standard deviation in parentheses.

## Data Availability

The data presented in this study are available on request from the corresponding author.

## Abbreviations

HCC, hepatocellular carcinoma; TACE, transarterial chemoembolisation; US, ultrasound; CT, computed tomography; MRI, magnetic resonance imaging; EASL, European Association for the Study of the Liver; HVPG, hepatic venous pressure gradient; SD, standard deviation; NASH, nonalcoholic steatohepatitis; NAFLD, nonalcoholic fatty liver disease.
